# Clinicopathological Features and Postoperative Complication Management of a Postauricular Granular Cell Tumor: A Case Report

**DOI:** 10.1002/ccr3.72835

**Published:** 2026-06-01

**Authors:** Haotong Yuan, Chunhong Wang, Luran Wang, Dongxu Liu, Li Gao

**Affiliations:** ^1^ Department of Oral and Maxillofacial Surgery The Second Affiliated Hospital of Harbin Medical University Harbin China

**Keywords:** differential diagnosis, granular cell tumor, MRSE, postauricular region, secondary intention healing

## Abstract

Postauricular granular cell tumors mimic parotid malignancies. Their resection disrupts fragile local microcirculation due to inherently thin skin and limited subcutaneous buffer, triggering ischemic necrosis and MRSE infection. Clinicians must prioritize functional tissue preservation and vigilant wound care to prevent these severe complications.

## 1 Introduction

Granular cell tumors (GCTs) are rare neuroectodermal soft tissue tumors, most commonly originating from Schwann cells [1]. Although GCTs occur in various sites—including the skin, oral cavity, breast, and gastrointestinal, respiratory, and urogenital tracts [2]—presentation in the postauricular region is uncommon [3]. The differential diagnosis for postauricular mastoid masses is broad. Common lesions include epidermoid cysts and lymphadenopathy. Because the anatomical location borders the parotid tail, clinicians must distinguish these masses from parotid‐derived tumors such as Warthin tumor, pleomorphic adenoma, and adenoid cystic carcinoma. Due to their rarity and nonspecific clinical features, GCTs are seldom prioritized in initial diagnostic considerations, which increases the risk of misdiagnosis or missed diagnosis [4, 5].

Radiographically, GCT typically appears as an ill‐defined, infiltrative, multinodular hypoechoic or soft tissue mass [6]. In the postauricular region, imaging evidence of muscle and bone invasion—as demonstrated on MRI in this case—is frequently misidentified preoperatively as a parotid malignancy (such as adenoid cystic carcinoma or carcinoma ex pleomorphic adenoma), aggressive fibromatosis, or middle ear mastoid carcinoma. The diagnostic gold standard relies on histopathology and immunohistochemistry (IHC), specifically positive expression of S100, SOX10, and CD68. Intraoperative frozen section diagnosis remains particularly challenging. Due to the abundant eosinophilic granular cytoplasm, GCT cells on frozen sections can be misinterpreted as the oncocytes found in common benign parotid tumors (such as Warthin tumor or oncocytoma) or as a histiocytic reaction involving lipid‐laden macrophages [4]. Consequently, frozen section analysis alone is often insufficient for a definitive qualitative diagnosis; IHC is necessary to differentiate neurogenic lesions from epithelial‐derived tumors [7].

Therefore, the objective of this report is to present a rare case of a postauricular GCT complicated by postoperative MRSE infection, aiming to provide a clinical reference for the diagnostic workflow and the management of secondary complications in anatomically complex regions [5, 8].

## 2 Case History/Examination

A 54‐year‐old female presented with a right postauricular mass discovered over 10 years ago. The patient incidentally palpated the lesion, which was initially fingernail‐sized, with no obvious inciting factors. Following a period of slow growth to approximately the size of a duck egg, the mass demonstrated accelerated enlargement over the past year. Tenderness was elicited upon palpation, though no facial paralysis or other neurological deficits were present. The patient had no history of systemic treatment. Systemic functions, including appetite and bowel habits, were unremarkable. The patient had no history of smoking or alcohol consumption. Medical history was significant for hypertension managed long‐term with telmisartan. History of trauma, surgery, and blood transfusion was negative. Immunizations were complete, and no drug or food allergies were reported. The patient is married with healthy children. Family history was non‐contributory regarding hereditary or infectious diseases.

Systemic physical examination revealed no abnormalities. Specialist examination revealed slight maxillofacial asymmetry. A mass measuring approximately 3.0 × 3.5 cm was palpable in the right postauricular region. The lesion was firm and non‐mobile with relatively distinct boundaries. Tenderness was elicited upon palpation (+). The overlying skin color was normal. No symptoms of facial nerve involvement, auricular sensory changes, or hearing loss were identified. No significantly enlarged lymph nodes were palpated in the head or neck.

**FIGURE 1:**
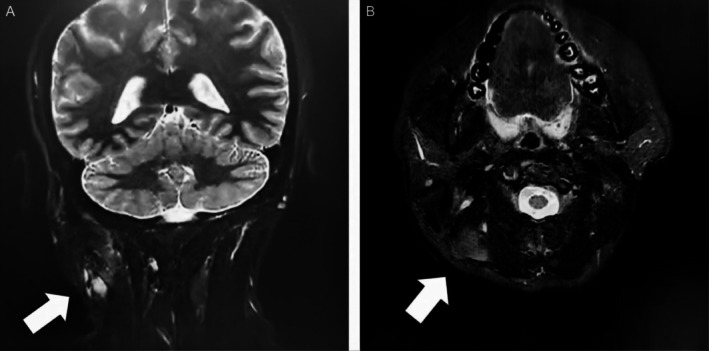
Preoperative Magnetic Resonance Imaging (MRI) assessment. (A) Coronal T2‐weighted fat‐saturated (T2WI Fat‐Sat) sequence shows soft tissue swelling in the right postauricular and mastoid region (white arrow). (B) Axial T2‐weighted fat‐saturated (T2WI Fat‐Sat) sequence demonstrates that, compared to the contralateral side, the right sternocleidomastoid and splenius capitis muscles are enlarged. The mass is closely apposed to the right occipital bone with signs of cortical irregularity, suggesting potential osseous invasion (white arrow).

Ultrasound examination was partially limited by surface scarring, which was secondary to chronic scratching induced by long‐term localized pruritus over the mass. A hypoechoic area measuring approximately 26 × 11 × 32 mm was identified in the right postauricular region, showing ill‐defined boundaries, irregular morphology, and heterogeneous internal echoes with no significant blood flow signal. To further evaluate the anatomical extent and peripheral soft tissue involvement, we performed a soft tissue MRI. Plain MRI revealed heterogeneous signals in the right postauricular and occipital soft tissues, characterized by patchy long T1 and long T2 signals that were hyperintense on fat‐suppression sequences. Focal circular long T2 signals suggested potential cystic degeneration (Figure 1). The right sternocleidomastoid and splenius capitis muscles were thickened with mildly elevated signals, likely reflecting tumor compression or an inflammatory response. Destruction of the right occipital bone surface was noted, while the mastoid appeared normal. Multiple nodular opacities were identified bilaterally in the neck, consistent with enlarged lymph nodes.

## 3 Methods (Differential Diagnosis, Investigations, and Treatment)

Given the 10‐year history and recent pain, we made a preliminary diagnosis of a right postauricular region mass of unknown nature. Clinical differential diagnosis presented significant challenges. Although the decade‐long course suggested benignancy, physical examination revealed a firm, tender mass, and MRI showed ill‐defined boundaries, focal cystic changes, and thickening of the adjacent sternocleidomastoid muscle. Given the contradictions between these infiltrative imaging features and the indolent clinical course, a definitive diagnosis distinguishing between rare benign entities and malignancies could not be reached via imaging alone. Surgical excision with histopathological and immunohistochemical analysis was necessary to confirm the diagnosis.

We performed the excision of the right postauricular mass under general anesthesia. We made an Ω‐shaped (Omega‐shaped) incision extending from the postauricular sulcus superiorly to the hairline. After incising the skin, subcutaneous tissue, and part of the platysma, we raised a skin flap to fully expose the mass. The lesion was situated on the surface of the occipital bone and mastoid, adjacent to the parotid gland and superior sternocleidomastoid muscle (SCM). The tumor was ill‐defined, firm, and solid, with poor vascularity and radial filamentous extensions, exhibiting a cartilage‐like consistency.

**FIGURE 2:**
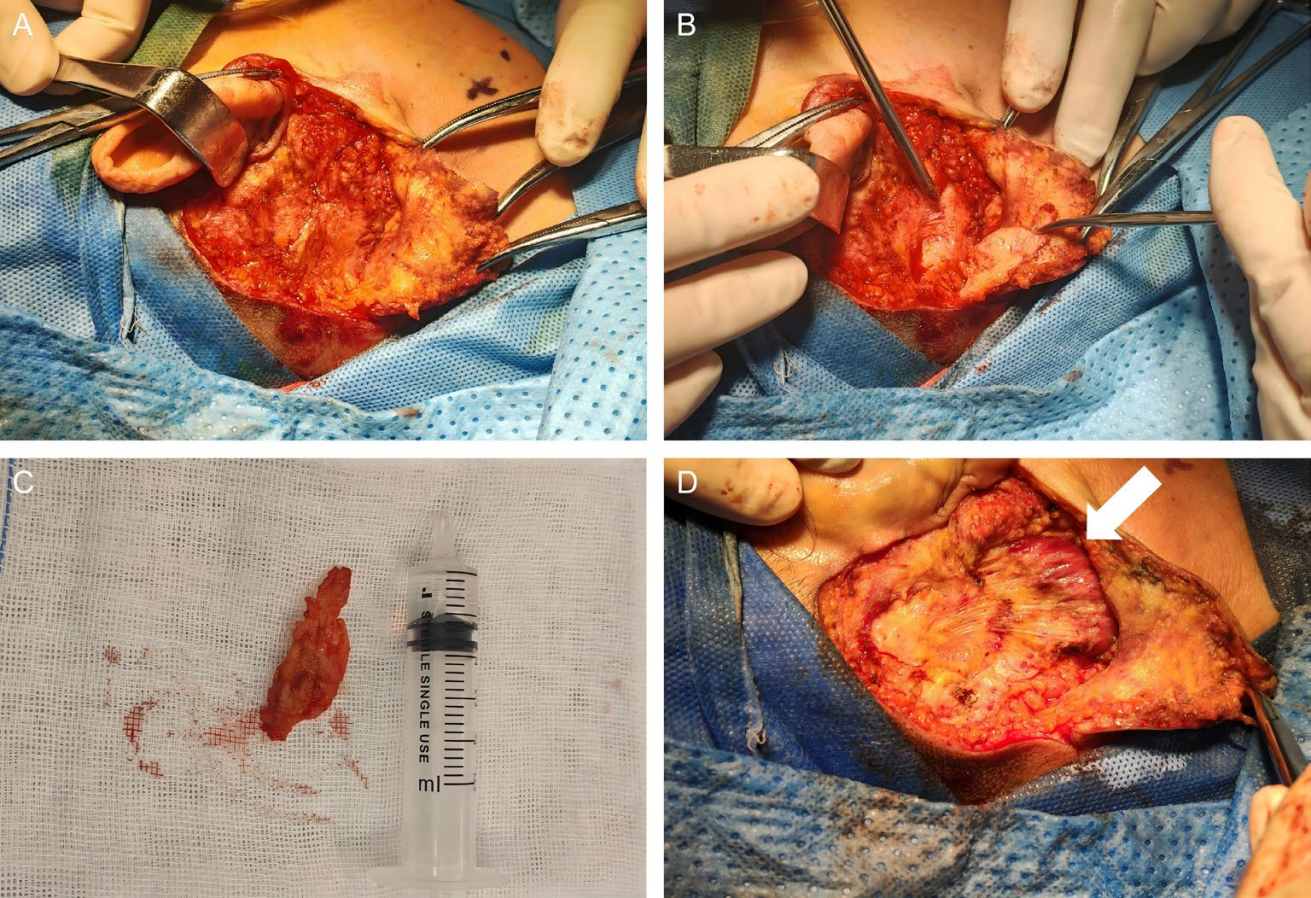
Intraoperative findings and gross specimen. (A) Exposure of the deep subcutaneous tumor in the right postauricular region. (B) Sharp dissection of the tumor from deep tissues, demonstrating ill‐defined margins. (C) Excised specimen characterized by an irregular, block‐like morphology and firm texture. (D) Viewing the surgical area of the tumor, the exposed sternocleidomastoid muscle (indicated by the white asterisk) can be seen extending in a radiating, filamentous manner.

Due to indistinct boundaries with surrounding tissues, we performed a layered excision (Figure 2). Intraoperative frozen section analysis revealed scattered pale‐staining cells within the myofibrous tissue, suggesting a potential granular cell tumor (GCT). Given the intimate connection between the tumor and the SCM, a deliberate subtotal resection was performed to preserve critical cervical motor function. Macroscopic tumor residue directly attached to the muscle was intentionally preserved to avoid postoperative impairment of neck rotation and lateral flexion. For recurrence surveillance, the patient is scheduled for a cervical ultrasound examination every 6 months. A secondary targeted surgical intervention on the SCM will only be considered if significant local tumor progression occurs or if the patient's daily functional activities become compromised. Postoperative pathology confirmed the diagnosis of GCT. Immunohistochemistry showed positive expression for S100, CD68, and SOX10, with a Ki‐67 index of approximately 2%. Markers for CK, EMA, and SMA were negative, consistent with the immunophenotype of GCT. Applying the accepted Fanburg‐Smith criteria, the tumor lacked malignant histological features: there was no evidence of tumor necrosis, spindling, vesicular nuclei with prominent nucleoli, increased mitotic activity, or a high nuclear‐to‐cytoplasmic ratio. Coupled with the low Ki‐67 proliferation index (~2%), the lesion was definitively classified as a benign granular cell tumor.

## 4 Postoperative Course and Complication Management

**FIGURE 3:**
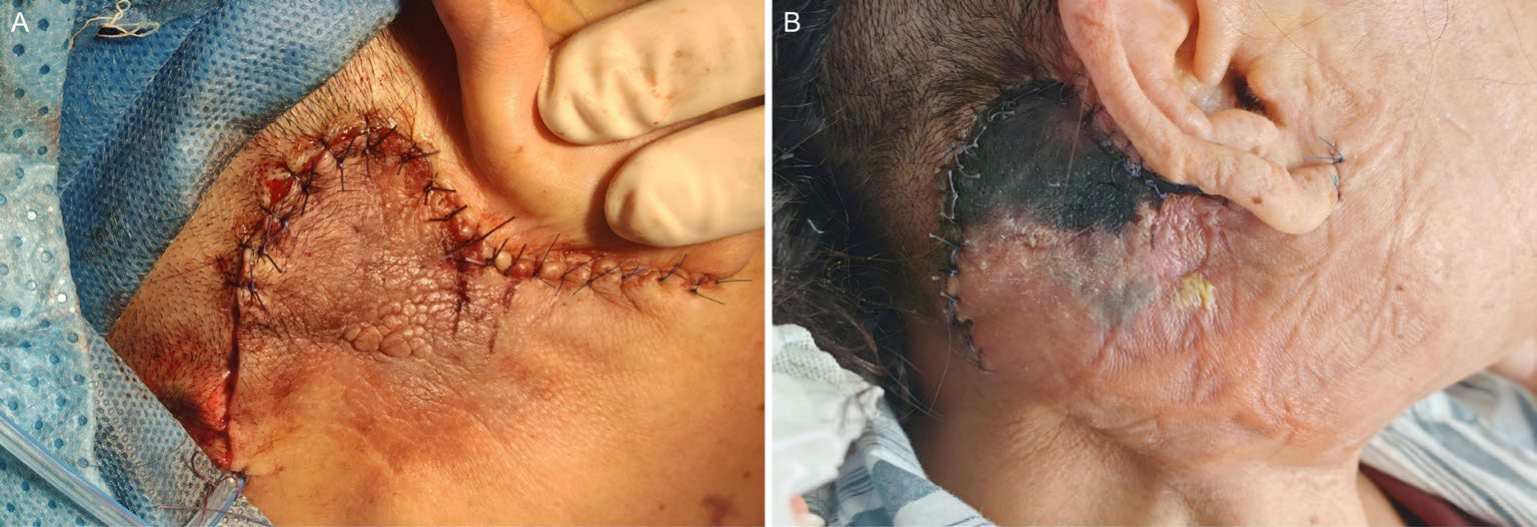
Postoperative wound healing and complications. (A) Immediate postoperative status; the incision is well‐approximated, and the skin flap appears pink with no signs of vascular compromise. (B) Postoperative day 6; the surgical site exhibits purplish‐black discoloration and eschar formation with surrounding erythema, indicating ischemia and secondary infection.

On postoperative Day 6, the surgical site exhibited significant erythema and swelling. The overlying skin showed signs of ischemia with a purplish‐black discoloration (Figure 3). Palpation revealed fluctuance, and needle aspiration yielded purulent discharge. We immediately performed incision and drainage to control the infection. The patient's vital signs remained stable, and her general status was unremarkable. Sutures were intact.

**FIGURE 4:**
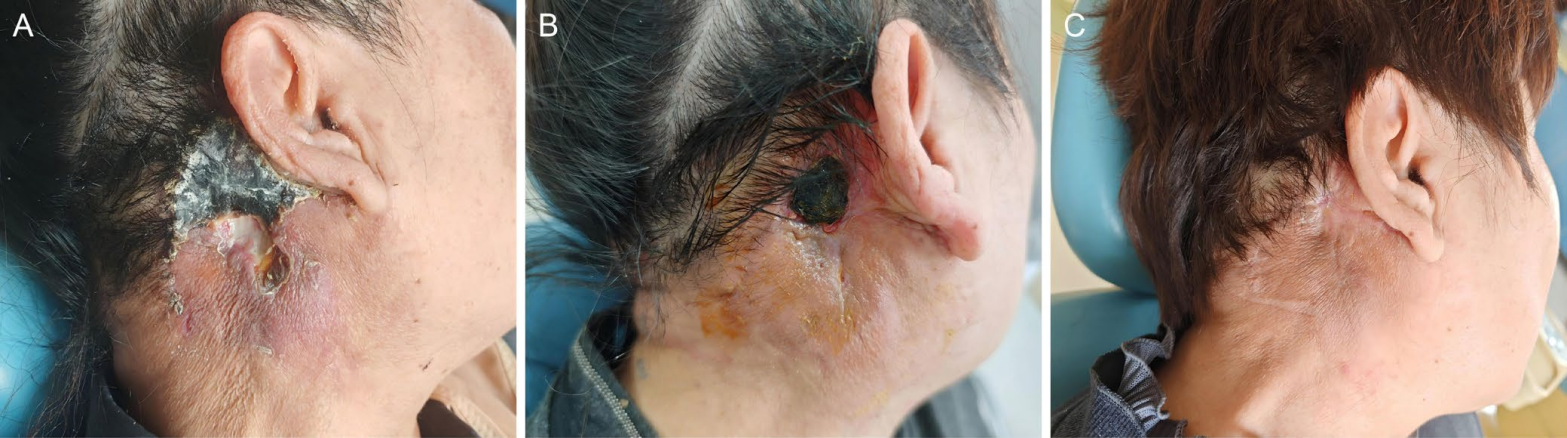
Postoperative wound healing evolution. (A) Postoperative day 25; the margins of the necrotic eschar are separating, demonstrating characteristic eschar separation and reflecting the delayed healing phase. (B) Postoperative Day 50; the wound shows significant contraction with fresh granulation tissue filling the base. A small area of residual black eschar remains centrally, illustrating a significantly prolonged secondary intention healing process secondary to compromised local blood supply. (C) Postoperative Day 97; the wound has achieved complete epithelialization with stable scarring and no signs of tumor recurrence, demonstrating a successful outcome of secondary intention healing.

Pus samples were submitted for bacterial culture and antimicrobial susceptibility testing (AST). Results identified the growth of *Staphylococcus epidermidis* . The AST indicated a multidrug‐resistant profile; a positive cefoxitin screen confirmed methicillin resistance. The strain demonstrated resistance to penicillin, oxacillin, erythromycin, clindamycin, and quinolones (levofloxacin and moxifloxacin) while maintaining sensitivity to vancomycin, teicoplanin, and daptomycin. Based on these findings, we diagnosed an opportunistic infection caused by methicillin‐resistant *Staphylococcus epidermidis* (MRSE). The antibiotic regimen was subsequently adjusted according to the AST results. Specifically, the patient received intravenous Linezolid (0.6 g q12h) for 1 day, followed by intravenous Tigecycline (50 mg q12h) for 3 days. Repeat source control involved localized debridement with iodophor, followed by iodoform gauze packing and elastic bandage compression. Following this targeted antibiotic therapy and active wound management, the systemic infection was effectively controlled. The patient remained afebrile, and the white blood cell count normalized. However, local wound healing was significantly protracted. By postoperative Day 25, the surgical site exhibited dry necrotic changes with a central thick black eschar beginning to separate from the margins. By Day 50, although the wound demonstrated marked contraction and was filling with fresh granulation tissue, a small area of residual necrotic eschar persisted centrally (Figure 4).

This prolonged healing process was attributed to compromised local blood supply in the postauricular region. Current management remains focused on conservative dressing changes with iodoform gauze to support healing by secondary intention. The patient remains under close follow‐up to monitor both the healing progression and potential local tumor recurrence. The detailed timeline of postoperative complications, wound care, and patient status at the last follow‐up is summarized in (Table 1).

**TABLE 1:** Timeline of postoperative complications, interventions, and wound healing progress.

Timepoint (Postoperative)	Clinical status and events	Intervention and wound care
Day 6	Erythema, swelling, purplish‐black discoloration, and fluctuance with purulent discharge	Incision and drainage; AST‐guided IV targeted antibiotics (Linezolid for 1 day, Tigecycline for 3 days)
Day 25	Systemic infection controlled. Surgical site exhibits dry necrotic changes with a separating central thick black eschar	Localized debridement with iodophor; iodoform gauze packing and elastic bandage compression
Day 50	Marked wound contraction. Fresh granulation tissue filling the base, with a small residual central eschar	Continued conservative daily dressing changes with iodoform gauze to support secondary intention healing
Day 97 (Figure 4C)	Complete epithelialization achieved. Wound is fully healed with stable scarring. No signs of tumor recurrence	Discontinue dressings. Routine clinical and ultrasound surveillance every 6 months

Abbreviations: AST, antimicrobial susceptibility testing; IV, intravenous.

## 5 Discussion

Granular cell tumors (GCTs) primarily derive from Schwann cells. Although GCTs occur in various anatomical sites, postauricular involvement is rare. Existing literature identifies adenomas, carcinomas, neurogenic tumors, and liposarcomas as the primary lesions in the postauricular region, with few reports specifically documenting GCT [9, 10]. Neurofibromas and schwannomas share overlapping imaging and intraoperative features with GCT, necessitating immunohistochemical (IHC) differentiation. Their differential diagnosis effectively leverages the positive expression of neural markers such as S100, SOX10, and CD68 [11, 12].

Compared to existing reports, the defining characteristics of this case are the 10‐year indolent period followed by recent rapid progression and the subsequent postoperative skin ischemia with secondary infection. Most literature focuses on the rarity of GCTs and diagnostic workflows, providing limited data on postoperative complications. Skin ischemia and infection following surgery in the postauricular and parotid regions are rarely documented; existing research predominantly focuses on facial nerve injury or tumor recurrence [13]. On postoperative day 3, the appearance of erythema, swelling, and purplish‐black discoloration, followed by abscess formation, underscored the precarious nature of the postauricular blood supply. The occurrence of this severe complication can be attributed to specific anatomical and surgical factors. The skin overlying the postauricular mastoid is intrinsically thin with limited subcutaneous tissue buffer. During the layered excision of the solid tumor, which adhered closely to the SCM, the inevitable disruption of the local subdermal perforating vessels occurred. This surgical trauma, combined with the postoperative tension on the skin flap, severely impaired local microcirculation. This localized ischemic environment not only induced eschar formation but also compromised regional tissue immunity, creating an ideal anoxic niche for the commensal *S. epidermidis* to colonize and transition into a multidrug‐resistant opportunistic pathogen. This complication emphasizes the necessity of preserving vascular integrity during resection in this anatomically complex region.

Bacterial culture of the purulent discharge confirmed a methicillin‐resistant *Staphylococcus epidermidis* (MRSE) infection. While *S. epidermidis* is typically a skin commensal [14], the localized microcirculation impairment and tissue trauma served as a catalyst for its transition into an opportunistic, multidrug‐resistant pathogen [15]. Managing such complex postoperative infections requires early pathogen identification and targeted antibiotic therapy—specifically vancomycin—based on antimicrobial susceptibility testing (AST) results rather than empirical treatment. This approach is critical for preventing the spread of infection. The successful management of this case provides a clinical reference for the early identification and intervention of skin complications following the excision of rare masses in the parotid and postauricular regions.

Diagnosing granular cell tumors (GCT) in the postauricular region remains challenging due to the significant overlap in clinical and radiographic features with common benign and malignant parotid tumors, which frequently leads to misdiagnosis. GCT typically presents as a slow‐growing, solitary mass with a firm consistency and irregular morphology. Radiographically, it often appears as a hypoechoic area lacking specific blood flow signals, making it difficult to distinguish from parotid adenomas, adenocarcinomas, or lipomas [16, 17]. In this case, MRI provided detailed soft tissue information, clearly demonstrating thickening and elevated signals within the sternocleidomastoid and splenius capitis muscle [18]. These radiographic findings correlate with the patient's preoperative tenderness and predicted the intraoperative challenges involving dense adhesions and indistinct boundaries between the tumor and adjacent musculature. During surgery, we observed that the mass was directly attached to the sternocleidomastoid muscle. We performed a subtotal resection at this site to avoid postoperative impairment of neck rotation and lateral flexion. This decision also considered the potential long‐term impact on cervical aesthetics and head posture, as well as the biological characteristics of GCT. Long‐term follow‐up is necessary to evaluate the outcomes of this surgical approach. This case underscores the importance of understanding the biological behavior of GCT and gaining clinical experience to improve the diagnosis and management of rare diseases.

Misdiagnosis as Warthin tumors, neurogenic tumors, or mastoid‐derived neoplasms is common, which often complicates surgical planning. Although typically an adult pathology, GCT can present in pediatric demographics, including rare congenital cases such as a granular cell mass in a newborn's alveolar ridge [19]. Such clinical diversity emphasizes that GCT should be considered regardless of patient age or anatomical site. Therefore, clinicians must emphasize the role of histopathology and immunohistochemistry (IHC). When imaging reveals atypical tissue involvement or when clinical findings are contradictory, obtaining early histological evidence is vital to prevent misdiagnosis and optimize surgical decision‐making [20, 21].

Definitive diagnosis of GCT relies on the specific expression of immunohistochemical (IHC) markers [22]. The tumor cells are characterized by abundant eosinophilic granular cytoplasm. Positive expression of neural markers—including S100, SOX10, and CD68—facilitates differentiation from parotid gland‐derived, myogenic, and other soft tissue tumors. Immunohistochemistry is essential for excluding malignancies with similar presentations, such as lymphoma or liposarcoma [23], particularly when the histomorphology is atypical or histological differentiation is poorly defined. In the present case, the co‐expression of S100, SOX10, and CD68 confirmed a Schwann cell origin, eliminating the risk of misdiagnosis as common solid neoplasms. Based on this case, clinicians must maintain high vigilance for unknown postauricular masses with a prolonged indolent period followed by rapid progression. Comprehensive preoperative imaging, multidisciplinary diagnostic workflows, and prompt intraoperative frozen section analysis with IHC are recommended to enhance diagnostic accuracy [24, 25]. Furthermore, individualized surgical margins and meticulous wound care protocols are essential to prevent severe postoperative ischemic and infectious complications [26].

## Author Contributions

**Haotong Yuan:** conceptualization, data curation, resources, writing – original draft. **Chunhong Wang:** formal analysis, validation. **Luran Wang:** investigation. **Dongxu Liu:** validation, visualization, writing – review and editing. **Li Gao:** conceptualization, methodology, supervision, writing – review and editing.

## Funding

This work was supported by the Natural Science Foundation of Heilongjiang Province (Grant No. PL2025H137).

## Ethics Statement

Ethical approval was not required for this case report in accordance with local guidelines.

## Consent

Consent Written informed consent was obtained from the patient to publish this report in accordance with the journal's patient consent policy.

## Conflicts of Interest

The authors declare no conflicts of interest.

## Data Availability

The data that support the findings of this study are available from the corresponding author upon reasonable request.
